# Synergistic effects of vagus nerve stimulation and antiseizure medication

**DOI:** 10.1007/s00415-023-11825-9

**Published:** 2023-06-27

**Authors:** Yaroslav Winter, Katharina Sandner, Martin Glaser, Dumitru Ciolac, Viktoria Sauer, Andreas Ziebart, Ali Karakoyun, Vitalie Chiosa, Assel Saryyeva, Joachim Krauss, Florian Ringel, Sergiu Groppa

**Affiliations:** 1 Department of Neurology, Mainz Comprehensive Epilepsy and Sleep Medicine Center, University Medical Center of the Johannes Gutenberg University Mainz, Langenbeckstr 1, 55131 Mainz, Germany; 2 Department of Neurology, Philipps-University, Marburg, Germany; 3 Department of Neurosurgery, University Medical Center of the Johannes Gutenberg University Mainz, Mainz, Germany; 4 Department of Neurology, Focus Program Translational Neuroscience (FTN), Rhine Main Neuroscience Network (rmn2), University Medical Center of the Johannes Gutenberg University Mainz, Mainz, Germany; 5 Department of Neurosurgery, University Hospital Mannheim, University of Heidelberg, Mannheim, Germany; 6 Department of Neurosurgery, Medical School Hannover, MHH, Hannover, Germany; 7 Laboratory of Neurobiology and Medical Genetics, Department of Neurology, Nicolae Testemitąnu State University of Medicine and Pharmacy, Chisinau, Moldova

**Keywords:** Epilepsy, Vagus nerve stimulation, Antiseizure medication, Synergistic effects

## Abstract

**Introduction:**

Vagus nerve stimulation (VNS) is an effective, non-pharmacological therapy for epileptic seizures. Until now, favorable combinations of different groups of antiseizure medication (ASM) and VNS have not been sufficiently addressed. The aim of this study was to identify the synergistic effects between VNS and different ASMs.

**Methods:**

We performed an observational study of patients with epilepsy who were implanted with VNS and had a stable ASM therapy during the first 2 years after the VNS implantation. Data were collected from the Mainz Epilepsy Registry. The efficacy of VNS depending on the concomitantly used ASM group/individual ASMs was assessed by quantifying the responder rate (≥ 50% seizure reduction compared to the time of VNS implantation) and seizure freedom (absence of seizures during the last 6 months of the observation period).

**Results:**

One hundred fifty one patients (mean age 45.2 ± 17.0 years, 78 females) were included in the study. Regardless of the used ASM, the responder rate in the whole cohort was 50.3% and the seizure freedom was 13.9%. Multiple regression analysis showed that combination of VNS with synaptic vesicle glycoprotein (SV2A) modulators (responder rate 64.0%, seizure freedom 19.8%) or slow sodium channel inhibitors (responder rate 61.8%, seizure freedom 19.7%) was associated with a statistically significant better responder rate and seizure freedom than combinations of VNS and ASM with other mechanism of action. Within these ASM groups, brivaracetam showed a more favorable effect than levetiracetam, whereas lacosamide and eslicarbazepine were comparable in their effects.

**Conclusion:**

Our data suggest that the combination of VNS with ASMs belonging to either SV2A modulators or slow sodium channel inhibitors could be optimal to achieve a better seizure control following VNS. However, these preliminary data require further validation under controlled conditions.

## Introduction

Vagus nerve stimulation (VNS) is an effective, non-pharmacological therapy for epileptic seizures [9, 10, 24, 26, 27, 31]. Several studies have already demonstrated the effectiveness of VNS in reducing the frequency of epileptic seizures with responder rates varying between 33 and 89% [5, 8, 13, 14, 23]. Despite the numerous and increasingly available pharmacological and non-pharmacological treatment options for patients with epilepsy, there are still many patients with persistent seizures and refractory epilepsy, which could benefit from neurostimulation [17, 25]. Since VNS is approved only as an adjunctive therapy for epilepsy treatment, all patients with VNS also receive antiseizure medication (ASM). As the number of epilepsy patients benefiting from VNS increases, it is still unclear which ASMs work in a synergistic manner with this type of neurostimulation. While the rational combination of different ASMs was an objective of previous research, the favorable effects of ASM in combination with VNS were beyond its scope [28]. The identification of ASMs having synergetic effects with VNS is of major importance. This finding would improve treatment response, reduce adverse effects, and increase the quality of life in patients with epilepsy [22, 32]. In addition, VNS could potentially reduce behavioral adverse effects of ASM due to its mood improving properties. Effectively, the knowledge of synergetic effects of VNS with a specific mechanism of action of ASM will optimize the therapeutic response and reduce the side effects of epilepsy treatment.

The aim of this study was to identify the favorable combinations of VNS and ASMs in terms of their efficacy.

## Methods

### Study design and clinical evaluation

In this observational study, we included patients with epilepsy who were implanted with VNS and did not change their ASM during the 2 years after the implantation. All patients were treated at the Comprehensive Epilepsy and Sleep Medicine Center, Germany, which is integrated into the Department of Neurology of the University Medical Center of the Johannes Gutenberg University Mainz, Germany. Implantation of VNS was performed in the neurosurgical departments of the three German university hospitals—University Medical Center Mainz, University Hospital Mannheim and Medical School Hannover. VNS implantation and post-operative management was carried out according to standard of care procedures. Data of the patients were retrieved from the Mainz Epilepsy Registry (MAINZ-EPIREG), which is focused on prospective evaluation of the disease course of patients with epilepsy. In order to estimate the combined efficacy of different ASMs and VNS, responder rate and seizure freedom were estimated for separate ASM groups. Responder rate was defined as the reduction in seizure frequency ≥ 50% per month compared to the time of VNS implantation. Seizure freedom was defined as the absence of seizures during the last 6 months of the observation period. The monthly seizure frequency at the study onset was calculated considering the 6 months preceding the implantation of VNS. The monthly seizure frequency at the end of the second year after the implantation of VNS was calculated considering the last 6 months of the observation period. The seizure frequency was recorded in a systematic way before the implantation of VNS and during the whole study period by means of standardized patients’ diaries. This study was approved by the local ethics committee and all of the patients have signed informed consent for participation in this study.

### Statistics

Statistical analysis was performed using IBM SPSS Statistics Version 23.0 (IBM Corp., Armonk, NY, USA). The Kolmogorov–Smirnov test was used to assess the distribution of data. Variables are presented as mean, standard deviation (SD) and range. A t-test or analysis of variance was applied for group comparisons of normally distributed variables. For non-normal distribution, the Mann–Whitney U-test (two independent groups) or the Kruskal–Wallis test (more than two independent groups) was used. Statistical significance was set to a p-value of < 0.05. Multiple regression analysis was performed to identify the independent factors that determine the responder rate and seizure freedom on stable therapy 2 years after VNS implantation.

## Results

One hundred fifty one patients (mean age 45.2 ± 17.0 years, 78 females) with epilepsy and VNS were included in the study. The duration of epilepsy before the VNS implantation was on average 17.2 ± 11.6 years. Data on demographics and clinical parameters are shown in Table 1. The following side effects due to VNS therapy were reported: postoperative pain in 13.9% of patients (n = 21), which disappeared within 3 weeks after the implantation, coughing in 9.3% (n = 14) of patients and hoarseness in 5.3% (n = 8) of patients. There were no specific side effects reported due to the combination of VNS and ASMs. In our study population, 36 (23.8%) patients did not benefit from the VNS treatment, being similar to existing literature.

**Table 1:** Data on demographics and clinical parameters of patients with vagus nerve stimulation

	All patients (n = 151)	Fast sodium channel inhibitors (n = 86)	Slow sodium channel inhibitors (n = 76)	SV2A modulators (n = 86)	AMPA antagonists (n = 58)	GABA agonists (n = 84)	Calcium channel inhibitors (n = 47)	CA inhibitors (n = 56)
Age, years								
Mean ± SD (range)	45.2 ± 17.0 (20–81)	44.6 ± 17.4 (21–81)	46.9 ± 17.3 (21–78)	44.7 ± 18.12 (20–81)	42.0 ± 16.7 (20–81)	43.1 ± 16.3 (20–78)	44.6 ± 16.9 (23–78)	44.6 ± 1 7.5 (20–78)
Gender, n (%)								
Male	68 (45.0)	35 (43.2)	30 (42.3)	35 (42.2)	29 (52.7)	34 (43.0)	26 (56.5)	26 (50%)
Female	83 (55.0)	46 (56.8)	41 (57.7)	48 (57.8)	26 (47.3)	45 (57.0)	20 (43.5)	26 (50%)
VNS type, n (%)								
SenTiva®	56 (37.1)	31 (38.3)	25 (35.2)	36 (43.4)	23 (41.8)	20 (25.3)	15 (32.6)	13 (25.0)
AspireSR®	76 (50.3)	38 (46.9)	38 (53.5)	44 (53.0)	29 (52.7)	50 (63.3)	17 (37.0)	26 (50.0)
Demipulse®	6 (4.0)	5 (6.2)	4 (5.6)	1 (1.2)	2 (3.6)	5 (6.3)	4 (8.7)	4 (7.7)
VNS Therapy® pulse model 102	13 (8.6)	7 (8.6)	4 (5.6)	2 (2.4)	1 (1.8)	4 (5.1)	10 (21.7)	9 (17.3)
Duration of epilepsy before VNS, years								
Mean ± SD (range)	17.2 ± 11.6 (2.2–58.2)	18.9 ± 12.2 (2.6–58.2)	16.4 ± 11.5 (2.2–58.2)	16.4 ± 10.1 (2.2–58.2)	17.6 ± 11.4 (2.5–48.3)	16.5 ± 10.3 (2.5–49.3)	16.8 ± 11.83 (2.8–45.7)	15.9 ± 12.2 (2.5–45.7)
Number of previous ASMs								
Mean ± SD (range)	4 ± 3 (0–14)	5 ± 3 (0–11)	4 ± 2 (1–9)	4 ± 3 (1–11)	5 ± 3 (1–11)	5 ± 3 (0–14)	5 ± 3 (0–14)	5 ± 3 (0–14)

*AMPA* α-amino-3-hydroxy-5-methyl-4-isoxazolepropionic acid, *ASM* antiseizure medication, *CA* carboanhydrase, *GABA* gamma-aminobutyric acid, *SV2A* synaptic vesicle glycoprotein 2A, *VNS* vagus nerve stimulation, *SD* standard deviation

Regardless of the used ASMs, the overall observed responder rate was 50.3% and seizure freedom after 2 years following the VNS implantation was 13.9%.

Multiple regression analysis showed that combination of VNS with synaptic vesicle glycoprotein (SV2A) modulators or slow sodium channel inhibitors was associated with a statistically significant better responder rate and seizure freedom than combinations of VNS and ASM with other mechanism of action. This analysis considered demographic data (age and gender), number of ASM in patient’s history, type of epilepsy and activated autostimulation of VNS. In addition to specific mechanism of action, the lesser number of previous ASMs was an independent factor of better responder rate and seizure freedom in patients with VNS in our study (Table 2).

**Table 2:** Multiple regression analysis of responder rate and seizure freedom in patients with vagus nerve stimulation

	Responder rate			Seizure freedom		
	B	95% CI	p-value	B	95% CI	p-value
Age	– 0.01	– 0.06; 0.04	0.71	0.01	– 0.09; 0.51	0.18
Female gender	– 0.06	– 0.22; 0.09	0.43	0.08	– 0.02; 0.19	0.12
Number of previous ASMs	**– 0.12**	**– 0.24; – 0.01**	**0.03**	**– 0.01**	**– 0.03; – 0.01**	**0.04**
Epilepsy type^a^	– 0.05	– 0.25; 0.08	0.41	– 0.02	– 0.13; 0.10	0.77
Autostimulation^b^	0.08	– 0.19; 0.11	0.37	0.04	– 0.11; 0.03	0.24
Fast sodium channel inhibitors	– 0.92	– 1.15: 0.12	0.58	– 0.13	– 0.24: 0.32	0.61
Slow sodium channel inhibitors	**0.08**	**0.01; 0.11**	**0.03**	**0.09**	**0.01; 0.13**	**0.02**
SV2A modulators	**0.22**	**0.07; 0.36**	** < 0.01**	**0.27**	**0.08; 0.54**	** < 0.01**
AMPA antagonists	– 0.13	– 2.82; 0.03	0.12	– 0.02	– 0.19; 0.11	0.81
GABA agonists	0.17	– 0.08; 0.87	0.11	0.06	– 0.11; 0.92	0.18
Calcium channel inhibitors	– 0.35	– 0.55; 0.14	0.49	– 0.02	– 0.19; 0.16	0.84
CA inhibitors	0.21	– 0.04; 0.72	0.57	– 0.23	– 0.19; 0.14	0.78
Constant	0.51	– 0.14; 0.89	0.21	0.20	– 0.10; 0,50	0.18

^a^Generalized vs focal

^b^Autostimulation (on = 1, off = 2)

*AMPA* α-amino-3-hydroxy-5-methyl-4-isoxazolepropionic acid, *ASM* antiseizure medication, *B* regression coefficient, *CA* carboanhydrase, *CI* confidence interval, *GABA* gamma-aminobutyric acid, *SV2A* synaptic vesicle glycoprotein 2A, *VNS* vagus nerve stimulation

Table 3 shows the responder rates and seizure freedom according to combination of VNS with different mechanisms of action and different ASM. The concomitant use of synaptic vesicle glycoprotein 2A (SV2A) modulators (64.0%) and slow sodium channel inhibitors (61.8%) was associated with the best responder rate (64.0% and 61.8%, respectively) and the best seizure freedom (19.8% and 19.7%, respectively). Within the group of SV2A modulators, brivaracetam showed a more favorable responder rate of 69.2% and seizure freedom of 21.2% as compared to levetiracetam (53.8% and 15.4%, correspondingly, p < 0.05). There was only a minor difference in responder rate and seizure freedom between eslicarbazepine and lacosamide (Table 3).

**Table 3:** Responder rate and seizure freedom of patients with vagus nerve stimulation depending on the adjunctive ASMs

	Number of patients n (%)	Responder rate^a^ n (%)	Seizure freedom n (%)
Substance group			
Fast sodium channel inhibitors	86 (57.0)	31 (36.0)	5 (5.8)
Slow sodium channel inhibitors	76 (50.3)	47 (61.8)	15 (19.7)
SV2A modulators	86 (57.0)	55 (64.0)	17 (19.8)
AMPA antagonists	58 (38.4)	22 (37.9)	5 (8.6)
GABA agonists	84 (55.6)	29 (34.5)	4 (4.8)
Calcium channel inhibitors	47 (31.1)	16 (34.0)	4 (8.5)
CA inhibitors	56 (37.1)	21 (37.5)	4 (7.1)
Adjunctive ASMs			
Valproate	41 (27.2)	13 (31.7)	1 (2.6)
Lamotrigine	64 (42.4)	23 (35.9)	4 (6.3)
Lacosamide	59 (39.1)	37 (62.7)	11 (18.6)
Levetiracetam	39 (25.8)	21 (53.8)	6 (15.4)
Brivaracetam	52 (34.4)	36 (69.2)	10 (21.2)
Carbamazepine	19 (12.6)	3 (15.8)	0 (0)
Eslicarbazepine	24 (15.9)	14 (58.3)	4 (16.7)
Oxcarbazepine	10 (6.6)	3 (30.0)	0 (0)
Topiramate	22 (14.6)	8 (36.4)	1 (4.5)
Zonisamide	39 (25.8)	13 (33.3)	4 (10.3)
Perampanel	39 (25.8)	14 (35.9)	4 (10.3)
Pregabalin	11 (7.3)	2 (18.2)	0 (0)
Clobazam	21 (13.9)	4 (19.0)	2 (9.5)
Ethosuximide	9 (6.0)	2 (22.2)	0 (0)
Phenobarbital	11 (7.3)	4 (36.4)	0 (0)

*AMPA* α-amino-3-hydroxy-5-methyl-4-isoxazolepropionic acid, *ASM* antiseizure medication, *CA* carboanhydrase, *GABA* gamma-aminobutyric acid, *SV2A* synaptic vesicle glycoprotein 2A, *VNS* vagus nerve stimulation

^a^Responder defined as a patient with a reduction in seizure frequency of ≥ 50% compared to baseline

## Discussion

Although VNS was established as an effective non-pharmacological therapy for patients with epilepsy decades ago, the evidence on favorable combinations of VNS and ASMs is limited. The responder rate and seizure freedom observed in our study population were similar to the data from previous reports [5, 8, 14, 26, 27]. Despite this, some of the previous studies showed a lower rate of seizure freedom [11]. This difference could be explained by the fact that we defined seizure freedom as absence of seizures during the last six months of the observation period compared to other studies that adopted a longer period for seizure freedom definition.

Overall, studies have identified several factors that could be beneficial for VNS therapy. It seems to be particularly effective in patients that have a shorter duration of epilepsy before the VNS implantation, in patients that predominantly experience generalized seizures and in those patients with a low seizure frequency before the implantation [1, 6, 7, 18]. However, the available evidence on factors associated with the improved outcome following VNS in the treatment of epilepsy is rather heterogeneous and has not yet addressed the synergistic effects of VNS and pharmacotherapy [1, 5]. Interestingly, there is growing evidence on synergistic effects of VNS and ketogenic diet showing that the seizure frequency is reduced more during their combination as compared to when either one is used alone [2].

Overall, VNS therapy reduces pharmacological burden, exhibits no interactions with ASM and favors the reduced costs of pharmacotherapy [16]. The knowledge regarding possible synergistic effects of VNS and ASMs would help to identify the most effective therapeutic combinations and improve the outcome in epilepsy patients. However, we have found only one study investigating the effects of combination of VNS with different ASMs. Labar et al. were following patients on different ASMs during the 12 month period after the VNS implantation [19]. The following ASMs were evaluated in this study: levetiracetam, zonisamide, oxcarbazepine, carbamazepine, lamotrigine, valproate, topiramate, and phenytoin [19]. Unfortunately, the authors could not find any favorable combination of ASM with VNS in this study [19]. The study was performed nearly a decade ago and ASMs that have showed positive effects in our analysis were not established at that time (except for levetiracetam). In addition, the observation of 12 months is quite a short time period for the estimation of VNS therapy. During the first year after the implantation, there is a need for titration and optimization of VNS parameters, so the therapeutic effects may vary. We chose a longer observational period of 24 months in order to estimate the effects of a stable VNS therapy.

The pathophysiological explanation of the possible synergistic effects between VNS and SV2A modulators remains unclear. On the cellular level of VNS mechanism of action, it has been shown that VNS mediates GABA-ergic, serotonergic and noradrenergic transmission [3, 4]. Hence, a synergistic effect between VNS and GABA-ergic ASMs would be expected. Our clinical data could not prove this assumption, suggesting that VNS could involve the same neural pathways as GABA-ergic medication, without providing any additional benefit in combination. Another known mechanism of action of VNS is desynchronization of electroencephalography (EEG) rhythms [29]. Interestingly, a similar desynchronizing effect has been shown for levetiracetam in an experimental setting [20, 21]. Therefore, it could be speculated that SV2A modulators work in a synergistic manner with VNS by amplifying the desynchronization of pathological EEG rhythms. Due to its higher affinity for SV2A, brivaracetam probably has a more prominent effect on EEG desynchronization than levetiracetam, however it should be investigated in future experimental studies [15]. It well is known that valproate, carbamazepine, and clonazepam can suppress neuronal hypersynchrony [20, 21]. However, in their study, Niespodziany et al. showed that levetiracetam is distinct from these classical ASMs by its selective effect on collective neuronal responses, rather than on single neuronal activities, suggesting a novel desynchronizing effect of this drug [21]. Perhaps, VNS may potentiate this specific desynchronizing effect of levetiracetam.

Another important finding of our work is the possible reduction and prevention of side effects of ASM polytherapy. It is known that sodium channel blockers given in high doses or in cases of their combination produce such side effects as dizziness, somnolence, vomiting and diplopia [12, 30, 33]. Considering the mechanism of action of sodium channel blockers, which showed to be favorable in combination with VNS, epileptologists can avoid higher doses or irrational combinations of sodium channel inhibitors. However, the prevention of pharmacological side effects was out of the scope of our study because we included patients on stable medication. Our findings would encourage further studies on the reduction of side effects of pharmacotherapy by employing an optimal combination of VNS and ASMs.

Among the limitations of this study was its observational design implying that the evidence could not be provided at the level of randomized control studies. The subgroups of patients treated with oxcarbazepine, pregabalin, ethosuximide and phenobarbital were small and should be addressed in larger studies. In addition, phenytoin, gabapentin, cannabidiol, fenfluramine and cenobamate were not included in this analysis and should also be considered in future studies. While phenytoin and gabapentin are old generation ASMs and are seldom prescribed for long-term therapy, cannabidiol and cenobamate are new generation ASMs and their role in epilepsy treatment is a matter of increased interest. Also, we did not report the side effects of VNS in combination with ASMs in detail since we have not found any specific side effects due to the combination of VNS, and ASMs and only the side effects of VNS were reported. Next, in our study, we considered only patients who did not change their ASM after the VNS implantation that may constitute a selection bias. In our center, we motivate the patients not to change their ASM in first years after the VNS implantation by explaining that the improvement in seizure control occurs on a long-term. Nevertheless, we compared the clinical characteristics of patients excluded from the study due to changes in ASM (reduction or increase in number of ASM) with the study participants and did not find any significant differences in demographical or clinical (i.e. number of ASM or seizure frequency) parameters at study baseline, thereby mitigating the possibility of selection bias.

In conclusion, our data suggests that the combination of VNS with SV2A modulators or slow sodium channel inhibitors could be favorable to optimize seizure control. However, these findings should be investigated further under controlled conditions in order to provide a higher level of evidence.

## Data Availability

Original data can be provided by the corresponding author on demand.

## Abbreviations

AMPA: α-Amino-3-hydroxy-5-methyl-4-isoxazolepropionic acid
ASM: Antiseizure medication
CA: Carboanhydrase
EEG: Electroencephalography
GABA: Gamma-aminobutyric acid
MAINZ-EPIREG: Mainz Epilepsy Registry
SV2A: Synaptic vesicle glycoprotein 2A
VNS: Vagus nerve stimulation
