# Correction to: Synergistic effects of vagus nerve stimulation and antiseizure medication

**DOI:** 10.1007/s00415-023-12019-z

**Published:** 2023-10-17

**Authors:** Yaroslav Winter, Katharina Sandner, Martin Glaser, Dumitru Ciolac, Victoria Sauer, Andreas Ziebart, Ali Karakoyun, Vitalie Chiosa, Assel Saryyeva, Joachim K. Krauss, Florian Ringel, Sergiu Groppa

**Affiliations:** 1 Department of Neurology, Mainz Comprehensive Epilepsy and Sleep Medicine Center, University Medical Center of the Johannes Gutenberg University Mainz, Langenbeckstr 1, 55131 Mainz, Germany; 2 Department of Neurology, Philipps-University, Marburg, Germany; 3 Department of Neurosurgery, University Medical Center of the Johannes Gutenberg University Mainz, Mainz, Germany; 4 Department of Neurology, Focus Program Translational Neuroscience (FTN), Rhine Main Neuroscience Network (rmn2), University Medical Center of the Johannes Gutenberg University Mainz, Mainz, Germany; 5 Department of Neurosurgery, University Hospital Mannheim, University of Heidelberg, Mannheim, Germany; 6 Department of Neurosurgery, Hannover Medical School, MHH, Hannover, Germany; 7 Laboratory of Neurobiology and Medical Genetics, Department of Neurology, Nicolae Testemitąnu State University of Medicine and Pharmacy, Chisinau, Moldova

**Correction to: Journal of Neurology (2023) 270:4978–4984** 10.1007/s00415-023-11825-9

The original version of this article unfortunately contained a mistake. The corrected details are given below for your reading.

The authors’ name Joachim K. Krauss and Victoria Sauer were incorrectly written as Joachim Krauss and Viktoria Sauer.

The affiliation details for author Assel Saryyeva and Joachim K. Krauss were incorrectly given as “Department of Neurosurgery, Medical School Hannover, MHH, Hannover, Germany” but should have been “Department of Neurosurgery, Hannover Medical School, MHH, Hannover, Germany”’.

